# Myoinositol supplementation in the treatment of gestational diabetes mellitus: effects on glycaemic control and maternal-foetal outcomes

**DOI:** 10.1186/s12884-022-04852-3

**Published:** 2022-06-26

**Authors:** Valentina Guarnotta, Gianluca Cuva, Maria Pia Imbergamo, Carla Giordano

**Affiliations:** Dipartimento di Promozione della Salute, Materno-Infantile, Medicina Interna e Specialistica di Eccellenza “G. D’Alessandro” (PROMISE), Sezione di Malattie Endocrine, del Ricambio e della Nutrizione, Università di Palermo, Piazza delle Cliniche 2, 90127 Palermo, Italy

**Keywords:** Insulin requirement, Birth weight, Hypoglycaemia, NUtrition, Glucose intolerance

## Abstract

**Background:**

Gestational diabetes mellitus (GDM) is defined as glucose intolerance with onset during pregnancy. It is characterized by high risk of adverse outcomes for the mother and the foetus, if not adequately controlled. The aim of the study was to evaluate the effects of 4000 mg of myoinositol supplementation in women with GDM on maternal-foetal outcomes, compared to controls.

**Methods:**

A cohort of 330 women with GDM, 150 supplemented with myoinositol and 180 controls were enrolled. Clinical and metabolic parameters and the prevalence of maternal and foetal complications were assessed.

**Results:**

The same number of women in the two groups started insulin as additional therapy. Women treated with myoinositol more frequently had a long-acting insulin scheme of treatment than those untreated (*p*<0.001), while women untreated with myoinositol more frequently had a basal-bolus insulin regimen (p<0.001) compared to women on myoinositol.

Patients treated with myoinositol had significantly lower fasting plasma glucose (*p*=0.032), post-prandial dinner glucose (*p*=0.014), insulin requirement both in the 2nd and in the 3rd trimesters (*p*=0.001 and *p*<0.001, respectively), than those not treated with myoinositol.

With regard to maternal/foetal outcomes, lower birth weight (*p*=0.043) and frequency of hypoglycaemic events (*p*=0.001) were observed in women treated with myoinositol compared to controls.

**Conclusions:**

Women with GDM treated with myoinositol showed an improved glycaemic control in the 3^rd^ trimester of pregnancy and a lower insulin requirement, when insulin was added to the treatment, compared to controls. In addition, they showed lower preterm birth weight and neonatal hypoglycaemia, compared to women not supplemented with myoinositol.

## Background

Gestational diabetes mellitus (GDM) is defined as glucose intolerance with onset during pregnancy [1]. The mean worldwide prevalence of GDM is 30% of pregnancies and it is higher when IADSPG criteria are used for GDM diagnosis [2, 3]. Generally, it is diagnosed during the second or third trimester of pregnancy and screening is performed between the 24^th^ and 28^th^ weeks of pregnancy. However, there are some controversies on this late diagnosis, and precocious screening at the first antenatal visit is strongly recommended, notably in women at high risk for GDM. The risk factors for developing GDM are obesity [Body mass index (BMI) ≥ 30 kg/m^2^], maternal age ≥40 years, ethnicity (Asian, Middle East, African-American, and Pacific Islanders), personal history of GDM, polycystic ovary syndrome, previous macrosomic baby or perinatal loss, familial history of diabetes mellitus, high fasting blood glucose values, use of steroids and multiple pregnancies [4]. GDM is associated with maternal and perinatal comorbidities including gestational hypertension, preeclampsia, caesarean delivery, foetal macrosomia and respiratory distress syndrome [5–7] and for this reason an early diagnosis and adequate management are necessary. In addition, GDM is associated with a high risk of developing overt diabetes mellitus in later years [8, 9].

Lifestyle interventions during pregnancy reduce the risks associated with GDM and improve the maternal-perinatal outcomes [10]. Management of GDM includes diet, physical activity and when necessary pharmacological interventions [11]. The pharmacological therapy consists in insulin treatment, as first line therapy. Rarely, the use of hypoglycaemic agents such as metformin and glyburide can be considered, because they can cross the placenta and cause some adverse effects in the offspring [12, 13].

Recently, myoinositol has emerged as a possible supplementary treatment in GDM. Myoinositol has been widely demonstrated to improve insulin resistance and for this reason it is a cornerstone of polycystic ovary syndrome treatment, but it has also shown favourable effects on fertility and in the prevention of GDM [14]. Due to the advantages in improving insulin resistance, it may represent an adequate treatment in women with GDM, due to the need of guarantee an adequate glycaemic control and reduce the risk of maternal/perinatal complications in these patients, avoiding the risk of hypoglycaemic events that patients may experience with insulin treatment. Few studies have been conducted on the efficacy of myoinositol in the management of GDM and the dose, frequency and timing of administration of myoinositol are currently not adequately defined [15].

The aim of this study is to compare women with GDM supplemented with myoinositol at the dose of 4000 mg/day with women not supplemented with myoinositol (controls), evaluating the effects on glycaemic control, the need for further pharmacological intervention and maternal-foetal outcomes.

## Methods

We conducted a prospective study on 330 pregnant women with GDM, followed at the Unit of Endocrinology, University of Palermo (Italy) from January 2018 to December 2020. Inclusion criteria were women with GDM aged more than 18 years. Exclusion criteria were twin pregnancies, women with T1DM on insulin therapy using continuous subcutaneous insulin infusion and women with T2DM, pre-existing renal or cardiac disease, and low diet compliance. The research team included the principal investigator and the sub-investigator and the nurse who performed blood analysis.

The diagnosis of GDM was made according to the International Association of Diabetes and Pregnancy Study Groups and World Health Organization between the 10^th^ and 29^th^ weeks of gestation [16, 17]. It was diagnosed when fasting glucose was ≥ 92 mg/dL before 16^th^ week of gestation or when at least one serum glucose level was more than the expected threshold of 92, 180 and 153 mg/dl for fasting, 1h and 2 h respectively, after an oral glucose tolerance test at 16-18 weeks or 24-28 weeks of gestation [17, 18]. Women were divided into two groups: women supplemented with myoinositol at the dose of 4000 mg/day and women not supplemented with myoinositol (controls). Detailed information were provided about myoinositol beneficial effects in preventing GDM and treating PCOS reporting the results of the published several studies. Controls rejected the treatment due to the lack of largest studies on myoinositol treatment in GDM. The dose of myoinositol was chosen according to the previous studies conducted in prevention of GDM [19]. Myoinositol was started at the diagnosis of GDM and continued until the delivery. This intervention was designed by our diabetologist research team.

During the first visit, a detailed medical history was collected for each patient, including previous obstetric history, recurrent pregnancy loss (RPL), family and personal history of chronic disease. Pregestational anthropometric parameters including weight, height and body mass index were registered. Fasting blood glucose, glycated haemoglobin and urine were assayed. Patients were instructed to follow a specific nutrition plan based on their pregestational BMI and weight. In addition, they were trained in self-monitoring of blood glucose (SMBG) using a glucose meter, with the instruction to perform a minimum of 4 daily measurements (before and 1 hour after meals), as previously reported [20]. The American Diabetes Association and the American College of Obstetricians and Gynecologist (ACOG) recommend similar glucose targets for women with pre-existing diabetes and GDM as follows: fasting glucose less than or equal to 95 mg/dL (5.28 mmol/L), 1 hour after eating less than or equal to 140 mg/dL (7.78 mmol/L) and 2 hours after eating less than or equal to 120 mg/dL (6.67 mmol/L) [11, 21]. For women with uncontrolled glucose values for at least 7 days, insulin therapy was started and the total daily insulin requirement was calculated. The insulin regimen was chosen based on blood glucose values. Basal and short-acting insulins were initiated in women with fasting or postprandial glucose values higher than above-mentioned targets, respectively. The dose was calculated according to the weight about 0.2-0.4 U/kg.

We defined women as underweight when pregestational BMI <18.5 kg/m^2^, normal weight when BMI was between 18.5 and 24.9 kg/m^2^, overweight when BMI was between 24.91-29.9 kg/m^2^ and obese when BMI was more than 29.9 kg/m^2^. HbA1c was repeated every three months.

The outpatient visits were twice-three times-weekly until delivery. Acute complications were recorded during follow-up: episodes of ketosis, ketoacidosis and maternal hypoglycaemic events. Mean fasting plasma glucose (FPG), postprandial breakfast glucose (PBG), postprandial lunch glucose (PLG) and postprandial dinner glucose (PDG) were recorded for each patient during the visits.

Pre-gestational arterial hypertension was defined with detection of systolic blood pressure values >140 mmHg; diastolic >90 mmHg and/or taking antihypertensive drugs before pregnancy. As maternal outcomes we evaluated pregnancy-induced hypertension defined by detection of systolic blood pressure values >140 mmHg and diastolic >90 mmHg after the 20^th^ week of gestation, preeclampsia defined by the combination of arterial hypertension and proteinuria >300 mg/24h, recurrent ketonuria and glycosuria, evaluated by dip-and-read test strip dipped into a morning urine sample and we defined abnormal an urinary result over 2+, pregnancy loss before the 24^th^ week of gestation, defined as spontaneous abortion, caesarean section and preterm delivery. Preterm birth was defined as completion of gestation before the 37^th^ week.

Perinatal/neonatal outcomes such as birth weight (grams and percentiles), birth length (cm and percentiles), large and small for gestational age were defined as birth weight <10^th^ and ≥90^th^ percentiles, respectively, neonatal hypoglycaemia defined as capillary blood glucose <3.3 mmol/L, hypocalcaemia, hyperbilirubinemia, neonatal intensive care unit admission and respiratory distress syndrome were also assessed.

Data collection involved the following steps: identification of issues and opportunities for collecting data, the creation of a data-collection plan, the actual collection of the data and the analysis and interpretation of data collected. The reliability of instruments used to measure blood parameters was assessed by test-retest reliability by an expert team. Quality data collectors had specifical technical skills including sorting data, creating statistical models, administering surveys, delivering presentations and reports, cleaning data, making corrections, and doing basic statistical work.

All patients provided written informed consent. The study protocol was approved by the Ethics Committee of the Policlinico Paolo Giaccone hospital.

### Statistical analysis

Sample size was calculated based on the mean prevalence of 30% GDM in the worldwide, with a confidence level of 95% and a margin error of 5%.

SPSS version 17 and MedCalc version 11.3 were used for data analysis. Baseline characteristics were presented as mean ± SD for continuous variables; rates and proportions were calculated for categorical data. Normality of distribution for quantitative data was assessed by the Shapiro-Wilk test. The differences between the two groups (myoinositol treated vs. not treated) were detected by the unpaired Student’s t test for continuous variables (after testing for equality of variance: Levene test) and by the χ2 test and Fisher’s exact test (when appropriate) for categorical variables.

The differences between the two groups with p-values less than 0.05 were considered statistically significant.

## Results

The clinical characteristics of women with GDM treated with myoinositol vs. untreated ones are shown in Table 1. At baseline women treated with myoinositol had a significant lower gestational age at GDM diagnosis (p=0.002) and a lower frequency of pregestational arterial hypertension (p= 0.037), compared to those not treated. Insulin therapy was started when FBG was over 95 mg/dL (5.28 mmol/L) and/or 1 hour post-prandial blood glucose over 140 mg/dL (7.78 mmol/L). The number of women who started insulin therapy was similar in the two groups (myoinositol vs. control) Table 1. However, we observed that in the myoinositol group women were more frequently treated with long-acting insulin scheme than controls (*p*<0.001), who by contrast were more frequently treated with basal-bolus scheme than the myoinositol group (*p*<0.001) Table 1.

**Table 1:** Maternal characteristics of women with GDM treated vs. not treated with myoinositol

	Patients treated with myoinositol ***N***= 150	Patients not treated with myoinositol ***N***= 180	***p***
	***Mean ± SD***	***Mean ± SD***	
Age at GDM diagnosis (years)	32.6 ± 5.78	32.1 ± 5.48	0.412
Pregestational BMI (Kg/m^2^)	28.8 ± 6.63	28.1 ± 6.37	0.266
Pregestational weight (Kg)	75.3 ± 18.2	73.2 ± 18.7	0.281
HbA1c at GDM diagnosis (%)	5.34 ± 0.48	5.35 ± 0.51	0.859
Oral glucose tolerance test at GDM diagnosis			
Fasting glycaemia (mmol/L)	95.7 ± 9.81	95.1 ± 10.9	0.770
Glycaemia after 1 hour (mmol/L)	150.7 ± 36.5	163.5 ± 52.1	0.172
Glycaemia after 2 hours (mmol/L)	115.8 ± 30.8	122.4 ± 37.6	0.364
Gestational age at GDM diagnosis (weeks)	21.7 ± 4.92	23.6 ± 4.93	0.002
Weight at delivery (Kg)	84.1 ± 17.2	81.7 ± 17.5	0.213
Gestational age at delivery (weeks)	38.4 ± 1.18	38.7 ± 0.99	0.105
Gestational age at start insulin therapy (weeks)	27.2 ± 5.13	27.7 ± 6.15	0.721
	***Subjects (%)***	***Subjects (%)***	
Pregestational arterial hypertension	3 (2%)	9 (5%)	0.037
History of poliabortivity	35 (23.3%)	42 (23.3%)	0.601
Primipara	45 (30%)	68 (37.7%)	0.288
Multipara	105 (70%)	82 (45.5%)	
BMI category			
Underweight (<18.5 kg/m^2^)	3 (2%)	3 (1.6%)	0.704
Normal weight (18.5-24.9 kg/m^2^)	44 (29.3%)	54 (30%)	0.235
Overweight (24.91-29.9 kg/m^2^)	52 (34.6%)	62 (34.4%)	0.412
Obese (>29,9 kg/m^2^)	51 (34%)	61 (33.8%)	0.817
Insulin treatment	52 (34.6%)	57 (31.6%)	0.723
• Long-acting insulin alone	31 (20.6%)	8 (4.4%)	<0.001
• Short-acting insulin alone	10 (6.6%)	11 (6.1%)	0.989
• Basal-bolus insulin	11 (7.3%)	34 (18.8%)	<0.001
Metformin treatment	5 (3.3%)	3 (1.6%)	0.744

Five women on myoinositol therapy and 3 in the control group experienced were treated with metformin, 3 of them because experienced hypoglycaemic events when insulin treatment was prescribed, while the other 5 rejected insulin therapy.

With regard to maternal/foetal outcomes, a lower frequency of neonatal hypoglycaemia (*p*=0.001) and a lower birth weight (*p*=0.043) were observed in the myoinositol group compared to controls Table 2.

**Table 2:** Maternal and neonatal/perinatal outcomes in women with gestational diabetes mellitus (GDM) treated vs. not treated with myoinositol

	Patients treated with myoinositol (***n***= 150)	Patients not treated with myoinositol (***n***= 180)	***P***
	***Subjects (%)***	***Subjects (%)***	
***Obstetrical outcomes***			
Gestational hypertension	9 (6%)	7 (3.8%)	0.150
Preeclampsia	9 (6%)	5 (2.7%)	0.137
Preterm delivery	8 (5.3%)	16 (8.8%)	0.055
Caesarean rate	40 (26.6%)	36 (20%)	0.156
Recurrent glycosuria	13 (8.6%)	16 (8.8%)	0.948
Recurrent ketonuria	35 (23.3%)	33 (18.3%)	0.263
	***Mean ± SD***	***Mean ± SD***	
***Neonatal and perinatal outcomes***			
Birth weight (g)	3241 ± 443	3361 ± 406	0.043
Birth weight percentiles	72.6 ± 12.6	75.4 ± 13.7	0.056
Birth length (cm)	49.6 ± 1.83	49.3 ± 1.87	0.426
Birth length percentiles	60 ± 16.6	59.2 ± 18.6	0.703
APGAR score	8.89 ± 0.91	9.13 ± 0.77	0.070
	***Subjects (%)***	***Subjects (%)***	
Large for gestational age (LGA)	13 (8.8%)	13 (7.2%)	0.592
Small for gestational age (SGA)	12 (8%)	25 (13.8%)	0.096
Hypoglycaemia	11 (7.3%)	36 (20%)	0.001
NICU stay	8 (5.3%)	5 (2.7%)	0.224
Hyperbilirubinemia	18 (12%)	12 (6.6%)	0.089
Respiratory distress syndrome	4 (2.6%)	3 (1.6%)	0.524

*NICU* neonatal intensive care unit

None suspended the treatment with myoinositol and no adverse events were registered during the treatment.

**Fig. 1:**
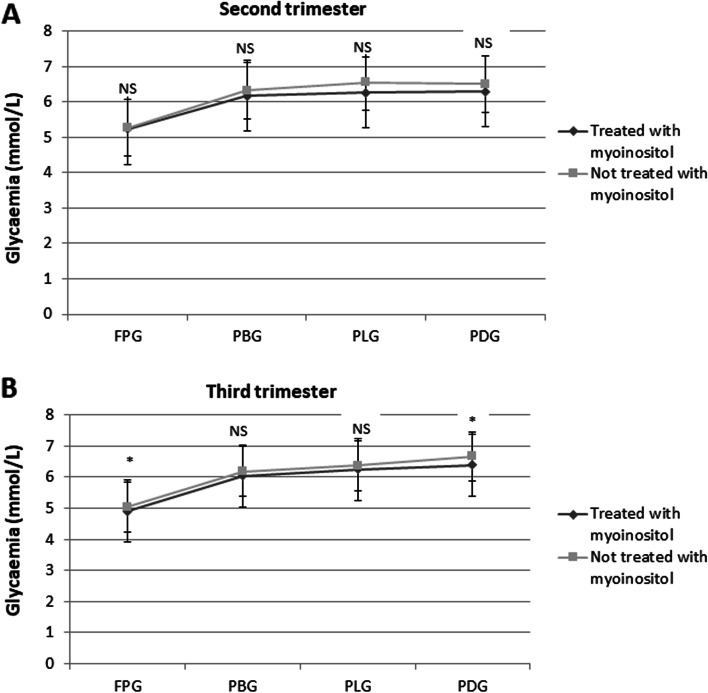
Mean fasting and postprandial glycaemia obtained from self-monitoring of blood glucose (SMBG) in women with gestational diabetes mellitus treated with myoinositol vs. untreated ones, in the second (**A**) and third trimesters (**B**). Data are presented as mean ± SD values. Student test was used to evaluate the differences between groups. FPG: fasting plasma glucose. PBG: postprandial breakfast glucose. PLG: postprandial lunch glucose. PDG: postprandial dinner glucose. NS: not significant. * *p*<0.05

**Fig. 2:**
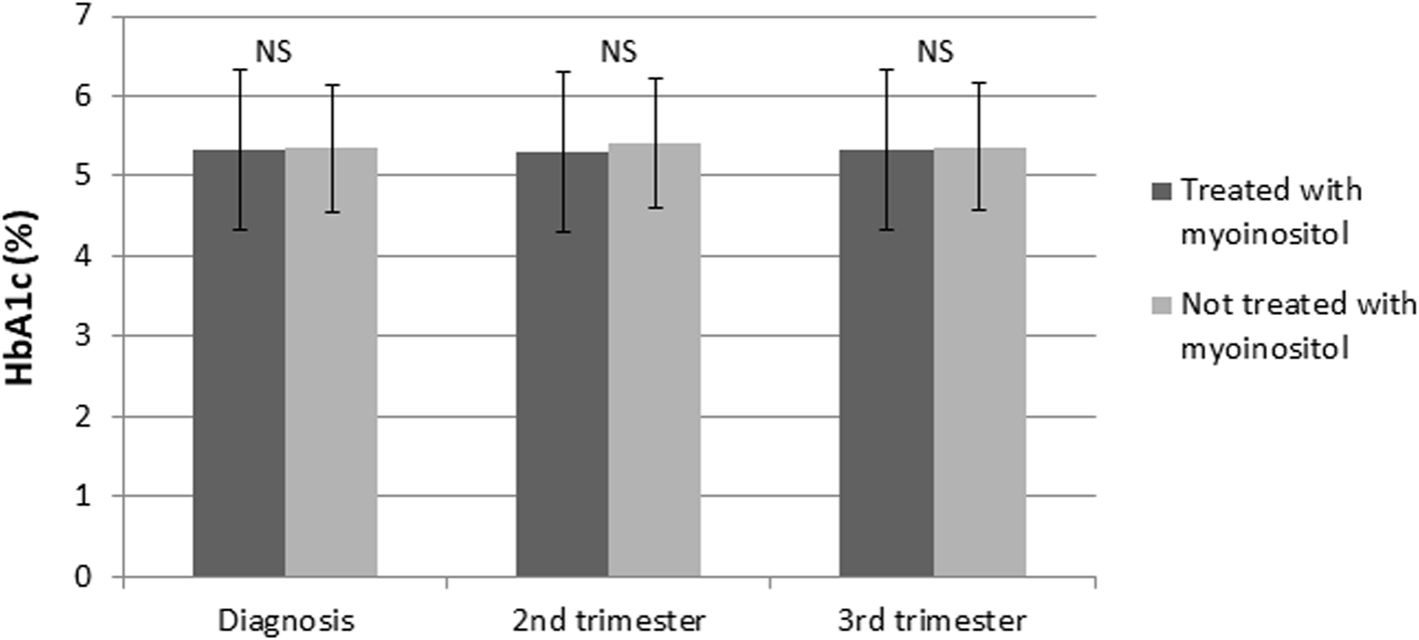
HbA1c at diagnosis and during the second and third trimesters in women with GDM treated with myoinositol vs. untreated ones. Values are mean ± SD. Student test was used to evaluate the differences between groups

**Fig. 3:**
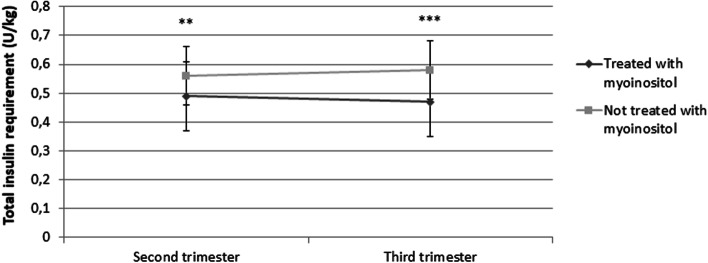
Total daily insulin requirements measured in units/kg during pregnancy (second and third trimester) in women with GDM with myoinositol treated vs. untreated ones, with the further addition of insulin therapy. **p <0.01; ***p <0.001. NS= not significant

In the 2^nd^ trimester no significant differences were observed in SMBG (Fig. 1 A) and HbA1c (Fig. 2) values between the two groups of women, while a lower insulin requirement was observed in women treated with myoinositol (*p*= 0.001) (Fig. 3).

In the 3^rd^ trimester women treated with myoinositol had significantly lower FPG (*p*=0.032), PDG (*p*=0.014) (Fig. 1) and insulin requirement (Fig. 3) (*p*< 0.001) compared to controls. No differences were observed in HbA1c values (Fig. 2).

We evaluated the effects of myoinositol in the two separate groups (women who were treated with additional insulin therapy and women who continued only diet), but we observed similar results (data not shown).

## Discussion

The current study is based on the hypothesis that high dose of myoinositol treatment may improve glycaemic control, reduce insulin requirement in women on insulin treatment and have a favourable effect on maternal/perinatal outcomes in women with GDM. This hypothesis is based on the positive effects of myoinositol treatment in the prevention of GDM and in the improvement of insulin resistance in women with PCOS.

Our data show that women with GDM supplemented with myoinositol at the dose of 4000 mg/day have better glycaemic control, evaluated by SMBG, have a lower insulin requirement when insulin is further added to the treatment and show low birth weight and low frequency of neonatal hypoglycaemic events compared to women not supplemented with myoinositol.

In the current study we found a similar need for additional insulin treatment in both patients with myoinositol and controls, with a different therapeutic scheme. Patients treated with myoinositol more frequently needed a long-acting insulin regimen than untreated ones.

Myoinositol has been widely shown to have a role in prevention of GDM and in the treatment of PCOS and fertility disturbances [14]. It has been reported to improve insulin sensitivity and to strengthen the endogenous insulin effect, exerting an insulin-like effect on metabolic enzymes by acting on the insulin-signal pathway [22, 23]. In addition, it improves fasting blood glucose and reduces glucose variability [24].

Most findings on myoinositol efficacy derive from studies conducted for the prevention of GDM, while a few studies have evaluated myoinositol as treatment for GDM.

In agreement with our results, Lubin et al. showed that women treated with 1200 mg/day of myoinositol had a significantly lower need for additional insulin treatment compared with untreated patients [25]. Similarly, Corrado et al. on 142 patients reported improved glucose and insulin values and HOMA-IR in patients treated with myoinositol at the dose of 4000 mg/day compared to controls [26]. Further, Fraticelli et al. compared 4 different schemes of treatment in patients with GDM, 400 mcg of folic acid alone, 4000 mg of myoinositol combined with 400 mcg of folic acid, 500 mg of d-chiro-inositol combined with 400 mcg of folic acid and 1100/27.6 mg myo/d-chiro-inositol plus 400 mcg folic acid in a total of 80 patients, showing that women treated with myoinositol had a decrease in HOMA-IR, a lower need for insulin treatment and lower birth weight [27].

In addition, a recent randomized study on 99 patients, 49 treated with myoinositol and 50 controls, showed better glycaemic control in patients on myoinositol treatment with a lower frequency of insulin addition, in line with our results [28].

With regard to maternal and neonatal outcomes, we found lower birth weight in women treated with myoinositol compared to controls, as also reported by Kulshrestha et al. [28]. Further, an interesting meta-analysis based on 5 trials aimed to evaluate the relationship between myoinositol and GDM showed lower birth weight and lower frequency of neonatal hypoglycaemia in the group with myoinositol treatment (at a dose ranging from 2000 to 4000 mg/day) [29], compared with controls, while no changes in macrosomia, respiratory distress syndrome, polyhydramnios, shoulder dystocia and preterm delivery complications were reported [29]. By contrast, a recent meta-analysis including 5 randomized controlled trials, aimed to evaluate the effects of myoinositol in prevention of GDM, showed a lower rate of GDM and a lower frequency of preterm delivery in patients treated with myoinositol than in those not treated [19].

Another recent randomized study compared the effects of combined myoinositol, ɑ-lactalbumin and folic acid vs. folic acid alone in a total of 120 women with GDM. This study, reported differently from the ours, a significant decrease in HOMA-IR and foetal growth, evaluated as abdominal circumference, and a lower frequency of preterm birth, while in line with our study showed reduced need of insulin in women treated with myoinositol compared to controls [30].

The present study has some limitations. First, our study does not have a randomized design and so the causal relationship between intervention and outcomes may be difficult to establish. Second, the myoinositol dose used is higher than that reported by other studies conducted in women with GDM, even though it is the same dose of the studies conducted for the prevention of GDM and for the treatment of PCOS. Third, some potential confounders including selection and recall bias cannot be excluded. Nonetheless, the strength of the study is the large sample of patients and the evaluation of insulin requirement and SMBG. For this reason, we believe that our findings have important implications for the clinical management and treatment of women with GDM.

## Conclusions

In conclusion, the current study shows that women with GDM treated with myoinositol show an improved glycaemic control in the 3^rd^ trimester of pregnancy and a lower insulin requirement, when insulin is added to the treatment, compared to controls. In addition, they show lower preterm birth weight and neonatal hypoglycaemia, compared to women not supplemented with myoinositol. Both women on diet and insulin treatment may have a beneficial effects from myoinositol supplementation in the management of glycaemic control and reduction of the risk of maternal/foetal complications.

Myoinositol may represent the first line therapy in women with a diagnosis of GDM. However, larger randomized studies should be performed to confirm our hypothesis.

## Data Availability

All data generated or analysed during this study are included in this published article
